# Effects of salt stress on interspecific competition between an invasive alien plant *Oenothera biennis* and three native species

**DOI:** 10.3389/fpls.2023.1144511

**Published:** 2023-03-21

**Authors:** Xiao Guo, Jin-Ye Ma, Le-Le Liu, Ming-Yan Li, Hui Wang, Ying-Kun Sun, Tong Wang, Kui-Ling Wang, Laura A. Meyerson

**Affiliations:** ^1^ College of Landscape Architecture and Forestry, Qingdao Agricultural University, Qingdao, China; ^2^ Institute of Ecology and Biodiversity, School of Life Sciences, Shandong University, Qingdao, China; ^3^ Department of Natural Resources Science, The University of Rhode Island, Kingston, RI, United States

**Keywords:** plant invasion, Oenothera biennis, functional traits, plant-plant interactions, salt stress

## Abstract

Biological invasions and soil salinization have become increasingly severe environmental problems under global change due to sea-level rise and poor soil management. Invasive species can often outcompete native species, but few studies focus on whether invasive alien species are always superior competitors under increasing stressors. We grew an invasive grass species, *Oenothera biennis* L., and three native grass species (*Artemisia argyi* Lévl. et Vant., *Chenopodium album* L., and *Inula japonica* Thunb.) as a monoculture (two seedlings of each species) or mixture (one seedling of *O. biennis* and one native species seedling) under three levels of salt treatments (0, 1, and 2 g/kg NaCl) in a greenhouse. We found that invasive *O. biennis* exhibited greater performance over native *C. album* and *I. japonica*, but lower performance compared to *A. argyi*, regardless of the soil salinity. However, salinity did not significantly affect the relative dominance of *O. biennis*. Interspecific competition enhanced the growth of *O. biennis* and inhibited the growth of *I. japonica*. Although *O. biennis* seedlings always had growth dominance over *C. album* seedlings, *C. album* was not affected by *O. biennis* at any salt level. At high salt levels, *O. biennis* inhibited the growth of *A. argyi*, while *A. argyi* did not affect the growth of *O. biennis*. Salt alleviated the competitive effect of *O. biennis* on *I. japonica* but did not mitigate the competition between *O. biennis* and the other two native species. Therefore, our study provides evidence for a better understanding of the invasive mechanisms of alien species under various salinity conditions.

## Introduction

Invasive alien species can have a profound and negative impact on the environment, biodiversity, ecosystem functioning, human health, and economy (Early et al., 2016; Pyšek et al., 2020; Rai and Singh, 2020; Diagne et al., 2021). Some intrinsic characteristics of the alien species may promote their invasiveness (van Kleunen et al., 2010). Since invasive alien species and native species occurring in the same habitats often face similar or even identical environmental selection pressures, the differences in functional traits between invasive and native species may be an important factor, influencing the successful invasion (Čuda et al., 2015; Wang et al., 2018). Two opposing hypotheses about trait values have been proposed to explain the success of alien species. The phenotypic convergence hypothesis is based on habitat filtering mechanisms and states that alien species that are more similar to native communities are more likely to succeed (Ordonez et al., 2010; Drenovsky et al., 2012). The phenotypic divergence hypothesis is based on limiting similarity and suggests instead that the differences in functional traits between invasive and native species contribute to the success of invasive species (MacArthur and Levins, 1967; Ordonez et al., 2010; Guo et al., 2020). An earlier meta-analysis suggested that invasive alien plant species generally have higher values than some native plants for traits reflecting physiology, size, and fitness (van Kleunen et al., 2010). However, another study showed that there were no significant trait differences between invasive species and native species (Lennox et al., 2015). Therefore, a universal conclusion has not been drawn about the success of alien invasive species relying on similarities or differences between invasive and native species.

Plant-plant interactions, including competition, have long been recognized as a key driver of community composition, structure, and dynamics (Yang et al., 2022), especially for determining the success of invasive species (Grainger et al., 2019). Invasive species are usually considered better competitors relative to natives for resources, space, and mutualists (Davis et al., 2000; Reinhart and Callaway, 2006; MacDougall et al., 2009). In addition, the importance of positive interactions (facilitation) in plant communities is also widely recognized (Brooker et al., 2008) and occurs when one species benefits from the presence of another (Brooker et al., 2008; Bimler et al., 2018; Grainger et al., 2019), for example *via* nutrient accumulation, stress mitigation, and protection from herbivores (Yang et al., 2022).

While there is extensive literature on the interactions between paired invasive alien and native species (Čuda et al., 2015; Legault et al., 2018; Pyšek et al., 2019; Hierro et al., 2022), pair-wise experiments using invasive and native plant species may be biased (Vilà and Weiner, 2004) because the selection of invaders and natives for the study is non-random and tends to favor highly competitive invaders and relatively less competitive natives (Zhang and van Kleunen, 2019). The resident community within the introduced range of the invasive species is composed of multiple native species with different functional traits, and invasive species usually face different competitive pressures from multiple native species (Zhang et al., 2020). However, so far few studies have been conducted to compare the effects of one invasive alien species on multiple native species in the same experiment, especially in the context of climate change.

Extensive studies have been conducted on the effects of salt stress on the morphology and physiology of invasive and native individually (Mao et al., 2016; Baniasadi et al., 2018; Liu et al., 2019). However, little research has focused on whether the competitive effects between invasive and native species are affected by salinity. According to the stress gradient hypothesis, interactions among plants are context-dependent, shifting from competition to facilitation as environmental stress increases (Bertness and Callaway, 1994). Soil salinity plays an important role in promoting or limiting the spread of invasive species and is a determining factor in colonization (Erfanzadeh et al., 2010; Rouifed et al., 2012) because, depending on the species and the salinity level, salinity tolerance constrains plant growth, resulting in significant changes in plant growth, morphological, and eco-physiological characteristics (Mao et al., 2016; Baniasadi et al., 2018; Sogoni et al., 2021). Further, salinity could influence invasion success, i.e., high salinity might increase the relative competitive ability of either native or invasive species depending on their tolerance (Al Hassan et al., 2016). Previous studies have demonstrated that salinity can enhance the replacement of native plants by invasive plants (Liu et al., 2015; Bollen et al., 2016; Legault et al., 2018), but salinity also can inhibit the establishment and performance of some invasive plants (Qi et al., 2017; Liu et al., 2019). Therefore, whether salinity can promote the invasion of exotic species is debatable.

*Oenothera biennis* L. (common evening primrose, Onagraceae) is an erect biennial native to eastern North America (Steckel et al., 2019). It is widely distributed in temperate regions around the world and is regarded as a severe invader in China, especially in coastal areas (Steckel et al., 2019; Zhang et al., 2021). *Oenothera biennis* preferentially occupies open and disturbed habitats (e.g. fields, roadsides, and lakeshores) (Puentes and Johnson, 2016), and severely damages the local natural ecosystem (Thijs et al., 2012). Previous studies have confirmed that high leaf area, the reproductive period in late summer, and long roots were key traits for the successful colonization of invasive *O. biennis* (Stanisci et al., 2010). Three native species, i.e., *Artemisia argyi* Lévl. et Vant., *Chenopodium album* L., and *Inula japonica* Thunb., were selected, as they are common native herb species in the eastern coastal region of China. *Artemisia argyi* is a perennial herb belonging to the family of Compositae that reproduces primarily by rhizome and small seeds (Ren et al., 2021). *Artemisia argyi* was chosen because it is widely distributed in the habitats inhabited by *O. biennis* and can become the dominant species in some native communities. *Chenopodium album* is an erect annual herb with characteristic goosefoot-shaped leaves and a strong taproot, belonging to the family of Chenopodiaceae. *Inula japonica* is a perennial herb belonging to the family of Compositae, which has lanceolate leaves and short rhizomes. Both *C. album* and *I. japonica* also coexist with *O. biennis* and share similar habitat types (i.e., coastal, farmland, along roads, and lakeshores) as *O. biennis*.

To assess the performance and competition effects of an invasive species on selected natives in response to increasing salinity, we conducted a greenhouse experiment using an invasive species, *Oenothera biennis* and three native species (*Artemisia argyi*, *Chenopodium album*, and *Inula japonica*). The seedlings of *O*. *biennis* and the three native species were planted in monocultures and species mixtures respectively. Three levels of salinity were applied to simulate increasing salt stress. The following questions were addressed:

(1) Is *O. biennis* generally a superior competitor to the three native species (*A. argyi*, *C. album*, and *I. japonica*) under mixed cultivation conditions? If so, what functional traits confer the competitive advantage on *O. biennis*?

(2) How does mixed culture affect the functional traits of invasive and native species compared to the monoculture?

(3) Does salt stress influence the biological interactions between the invasive species and three native species?

## Materials and methods

### Study site and plant materials

The experiment was conducted at Qingdao Agricultural University (36°31’N, 120°39’E), Qingdao city, Shandong Province, China. The site has a warm temperate monsoon climate, with an average annual temperature of 12.7°C, and the average annual precipitation is approximately 600−800 mm, most of which falls during the summer. The average temperature was lowest in January (monthly average temperature of - 2°C) and highest in August (monthly average temperature of 25.7°C). The experiment was conducted in the greenhouse at the experimental station to maintain a controlled and homogenous environment. The greenhouse was composed of steel tubes and its frame was covered by plastic film. By rolling up the plastic film on both sides, the greenhouse was kept well-ventilated.

The seeds of *O. biennis* were purchased from Huinong Seed Industry, Suqian City, Jiangsu Province, China on June 4, 2020, and then soaked in distilled water around 30−50°C for 24 h and stimulated to germinate by storing in a wet gauze tray during the early June of 2020. When the radicles of the seeds extended about 5 mm, they were transferred into a new tray. When the seedlings developed 3−4 true leaves, healthy and uniform seedlings were selected and transplanted into plastic pots (12 cm height × 14 cm diameter). The seedlings of *A. argyi* with 3-4 true leaves were obtained from Shouguang Xinxinran Horticulture Company, Weifang city, Shandong Province, China in late June 2020. Healthy and uniform growing seedlings of *C. album* and *I. japonica* with 3-4 true leaves were collected from the campus of Qingdao Agricultural University in late June 2020. Healthy and uniform seedlings of each species (*O. biennis*, *A. argyi*, *C. album*, and *I. japonica*) were selected and transplanted into plastic pots (12 cm height × 14 cm diameter) with two seedlings per pot on July 7, 2020. Each pot was filled with a mixture of 2:1:1 (v/v/v) loam, peat, and sand, which was carefully sieved through a 2 mm sieve to remove debris. The total weight of the substrate was 1 kg. During the experiment, water was added until the weight of the soil reached 70% of the filled capacity every day. Weeds and insects were controlled manually.

### Experimental design

The seedlings were arranged into seven cultivation types: monocultures with two seedlings of each species (*O. biennis*, *A. argyi*, *C. album*, or *I. japonica*) and mixtures with one seedling of *O. biennis* and one seedling of a native species (*A. argyi*, *C. album*, or *I. japonica*). Eventually, we obtained three types of mixtures: *O. biennis* mixed with *A. argyi* (Mixture1), mixed with *C. album* (Mixture2), and mixed with *I. japonica* (Mixture3). The range of salinity at the coastal shelter forests in Qingdao, China ranges from 1.2‰ to 2.3‰ according to our measurement of soil total salt using gravimetric method (DB37/T 1303-2009). Each cultivation type received three salinity treatments: 0, 1, and 2 g/kg NaCl dissolved in water (designated as S1, S2, and S3). The S1 treatment served as the control, and only water was applied. The S2 and S3 treatments were increased by 0.5g NaCl every week until reaching soil salt content. The S2 and S3 treatments were applied 2 and 4 times, respectively. The three salinity levels were chosen to simulate levels of soil salinity in the coastal shelter forests of Qingdao, China. The salt treatments started on July 17, 2020, and ended on September 17, 2020, lasting for 2 months. There were 21 treatment combinations (7 cultivation types × 3 salinity levels) in total and each combination contained ten pots as replicates. All pots were arranged randomly and rearranged regularly (7 days) during the experiment. During the experiment, mean temperature and mean relative humidity of greenhouse were 24.3 ° and 84.2%, respectively (DL-TH20, Hangzhou Gsome Technology Co., China).

### Trait measurements

Descriptions and units for the 16 functional traits measured in the experiment were shown in **Table 1**. The seedling height (H, from ground level to the terminal bud) and crown area (CA) were measured at the end of the salt treatment. The crown area was calculated as the formula of a diamond-shaped area (Eq. (1)), where “*a*” and “*b*” are lengths of the diagonal.

**Table 1:** Descriptions of traits and performance measures.

Abbreviation	Description	Units
H	Height	cm
CA	Crown area	cm^2^
TB	Total biomass	g
LBR	Leaf biomass ratio	
RSR	Root to shoot ratio	
Fv/Fm	Maximal quantum yield	
SLA	Specific leaf area	cm^2^ g^-1^
Chl a/b	Chlorophyll a/chlorophyll b	
Chl	Total chlorophyll content	mg g^-1^
LN	Leaf nitrogen concentration	mg g^-1^
LP	Leaf phosphorus concentration	mg g^-1^
LN/LP	The ratio of foliar nitrogen to phosphorus	
SN	Soil nitrogen concentration	mg g^-1^
SP	Soil phosphorus concentration	mg g^-1^
SN/SP	The ratio of soil nitrogen to phosphorus	
EC	Soil electrical conductivity	μs cm^-1^


(1) CA = 0.5 ×a×b.

Chlorophyll fluorescence parameters were measured with a Pocket PEA (Hansatech Instruments Ltd, King’s Lynn, UK) between 8:30 and 11:30 am on a sunny and cloudless day from 1 to 3 September 2020. Ten fully expanded leaves from the upper shoots were selected in each treatment (one leaf per pot for monocultures and one leaf per species per pot for mixture) and the measurements were conducted alternately among different treatments. Leaves were kept in the dark for 30 min to ensure complete relaxation of all reaction centers before measurements. The maximum quantum yield of photosystem II (*F_v_/F_m_*) was measured. Leaves of *C. album* were too small to measure chlorophyll fluorescence parameters.

Leaf morphology traits were measured from 5 to 7 September 2020. The third or fourth fully expanded leaves from the top per treatment were selected for measurement of the leaf area with a Yaxin-1241 portable leaf area meter (Yaxin Inc., Beijing, China). The leaves were first dried at 105°C for 0.5 h for deactivation of enzymes, and then oven-dried at 85°C for 48 h to calculate the leaf dry weight. Subsequently, the specific leaf area (SLA) was calculated as the SLA formula (Eq. (2)).


(2) SLA = leaf area/leaf dry weight.

Chlorophylls a and b were extracted and measured in mid-September 2020, using the ethanol method. Approximately 0.1 g of sheared fresh leaf tissue from the second or third leaf from the upper shoots in each treatment was soaked in 10 ml of 95% ethanol, and 8 replicates per treatment were selected. *Chenopodium album* had so few leaves that the concentration of chlorophyll could not be measured. After dark treatment for 24 h, the absorbance of the supernatant was determined at 649 nm (D649) and 665 nm (D665) using a UH5300 UV/VIS spectrophotometer (Hitachi, Inc., Tokyo, Japan). Chlorophyll a content (Chl a), chlorophyll b content (Chl b), total chlorophyll content (Chl), and Chlorophyll a/chlorophyll b (Chl a/b) were calculated separately as (Lichtenthaler, 1987):


(3) Chl a =(13.95×D665 −6.88×D6.49)×0.01/fresh weight of leaf tissue

(4) Chl b =(24.96×D649 − 7.32×D665)×0.01/fresh weight of leaf tissue

(5) Chl = Chl a + Chl b;

(6) Chl a/b = Chl a/Chl b.

At the end of the experiment, all seedlings were harvested, and the roots were washed carefully with tap water. Each whole seedling was separated into leaf, stem, and root. All seedling parts were first dried at 105°C for 0.5 h for deactivation of enzymes, and then oven-dried at 85°C for 48 h and weighed. Total biomass and biomass allocation were calculated as follows:


(7) Total biomass (TB) = leaf biomass + stem biomass + root biomass;

(8) Leaf biomass ratio (LBR) = leaf biomass/total biomass;

(9) Root to shoot ratio (RSR) = root biomass/aerial biomass.

The total biomass in monocultures was averaged from the two individuals in the same pot.

After the measurement of biomass, 3 to 8 leaf samples in each treatment were kept for the measurement of leaf nitrogen (LN) and phosphorus (LP) concentrations with the Kjeldahl method and the Molybdenum antimony-D-Isoascorbic-acid colorimetry method, respectively (Barbano et al., 1990). Then, we calculated the ratio of foliar nitrogen to phosphorus (LN/LP). Similarly, we measured soil nitrogen (SN) and phosphorus (SP) concentrations, as well as the ratio of soil nitrogen to phosphorus (SN/SP). In addition, soil electrical conductivity (EC) was measured with the ratio of water to the soil of 5:1 by using a conductivity meter (DDS-307A, LeiCi, Shanghai, China) and automatic temperature correction was set to a reference value of 25°C.

The relative dominance index (RDI) was used to assess the dominance of *O. biennis* in mixtures. The RDI was calculated according to Eq. (10).


(10) RDI=biomass of species/total biomass of two species in a pot 

(Yuan et al., 2013)

### Data analysis

For the RDI of *O. biennis* at each salinity level, all data were analyzed with a one-sample *t*-test to test whether these changes differed from 0.5. We calculated the log response ratio (ln *R*) as the effect size in each of the twelve functional traits to quantify the relative difference of different functional traits between invasive (*O. biennis*) and native species (*A. argyi*, *C. album*, and *I. japonica*) under three salinity treatments in the mixture. The log response ratio was calculated as ln *R* = ln (Trait*_I_*/Trait*_N_*), where Trait*_I_* stands for trait values of invasive species in the mixture, and Trait*_N_* represents trait values of native species in the mixture. A negative value of ln *R* indicates the trait favors native species whereas a positive value indicates the trait favors invasive species. Significant effect sizes are indicated by confidence intervals that do not overlap zero.

To better detect the effect size of biological interactions, the values of the natural log of relative yield ln (*RY*) in functional traits of the indices were calculated as ln (*RY*) = ln (Y*_ij_*/Y*_ii_*) (Keddy et al. 1994), where Y*_ij_* stands for the value of the traits of species *i* in the mixture treatment, and Y*_ii_* represents mean trait values of species *i* in the monoculture treatment. Ln (*RY*) > 0 means that species *i* responds positively to competition with species *j*. Ln (*RY*) < 0 means that species *i* responds negatively to competition with species *j.* Bias-corrected 95% confidence intervals (CIs) were calculated for each effect size. If the 95% CI did not overlap with zero, the effects were significant at *P* < 0.05 (Gómez-Aparicio, 2010). All statistical analyses were carried out with SPSS statistics 21.0 (SPSS Inc., Chicago, USA). All figures were drawn with the Origin 9.0 software (OriginLab Co., Massachusetts, USA). To test for differences in performance between plant species, among salinity treatments, and between cultivation types, a principal component analysis (PCA) was used by Origin 9.0 software (OriginLab Co., Massachusetts, USA). Traits that explained at least 30% of PC1 or PC2 were regarded as the main influence factors; these were H, CA, TB, LBR, RSR, F_v_/F_m_, SLA, Chla/b, Chl, LN, LP and LN/LP (**Table S1**).

## Results

### Relative dominance and competitiveness comparisons

**Figure 1:**
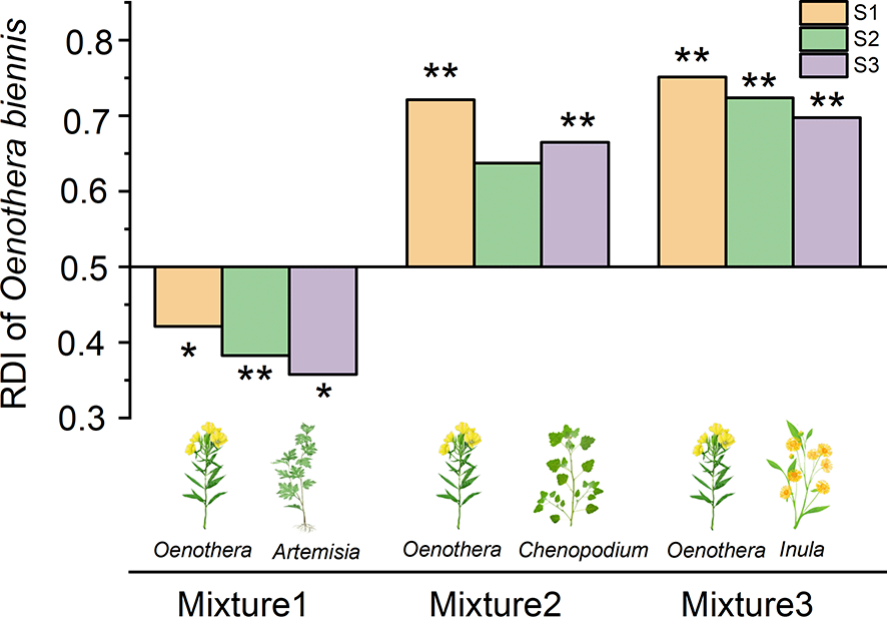
Relative dominance index (RDI) of *Oenothera biennis* in mixtures at three salinity treatments. *Oenothera biennis* in mixed culture with *Artemisia argyi* (Mixture1), *Chenopodium album* (Mixture2), and *Inula japonica* (Mixture3). Values are presented as 0.5 ± *SE* (*n* = 10 for all treatments). Significant effects are indicated by asterisks: **p ≤ 0.01 and *p ≤ 0.05.

The relative dominance index of *O. biennis* was higher than that of *C. album* and *I. japonica* in all salinity levels, while lower than that of *A. argyi* (**Figure 1**). No matter which native species it was mixed with, the RDI of *O. biennis* was not significantly affected by salinity (Mixture 1: *F* = 0.872, *p* = 0.433; Mixture 2: *F* = 0.979, *p* = 0.392; Mixture 3: *F* = 0.740, *p* = 0.489).

**Figure 2:**
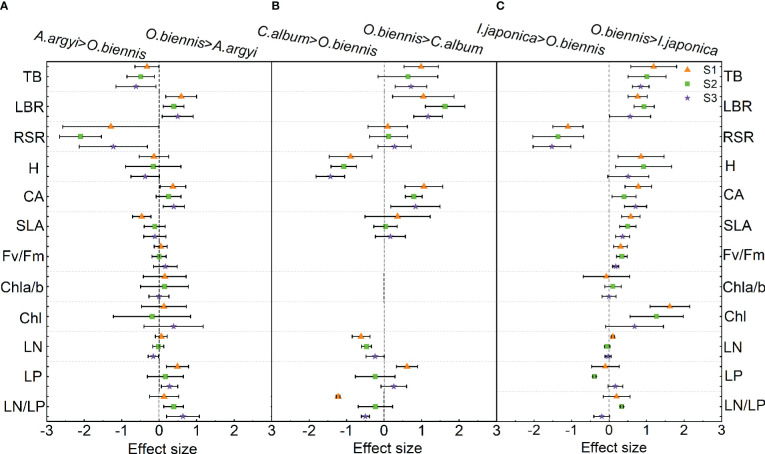
Relative difference values of different functional traits between invasive (*Oenothera biennis*) and native species (*Artemisia argyi*, *Chenopodium album*, and *Inula japonica*) under three salinity treatments in the mixture. The orange triangle illustrates the S1 treatment (control), the green square illustrates the S2 treatment (low salt treatment), and the purple pentagram illustrates the S3 treatment (high salt treatment). The capital letters represent the invasive species *O*. *biennis* and their three native species – *A. argyi* **(A)**, *C. album* **(B)**, and *I. japonica* **(C)** under the mixed cultivations. Error bars are 95% confidence intervals. Significant effect sizes are indicated by confidence intervals that do not overlap zero. Abbreviations have identical meanings as described in **Table 1**.

In the comparison of functional trait differences between *O. biennis* and *A. argyi*, four out of the twelve traits were significantly changed under S1 and S2 treatments, and six out of the twelve traits were significantly changed under S3 treatment (**Figure 2A**). Under S1 treatment, the LBR, CA and LP of *O. biennis* were higher than those of *A. argyi*, while the SLA was lower than that of *A. argyi* (**Figure 2A**). Under S2 treatment, LBR and LN/LP of *O. biennis* were higher than those of *A. argyi*, while TB and RSR were lower than *A. argyi* (**Figure 2A**). Under S3 treatment, LBR, CA, LP, and LN/LP of *O. biennis* were higher than those of *A. argyi*, while TB and RSR were lower than those of *A. argyi* (**Figure 2A**).

When comparing functional traits between *O. biennis* and *C. album*, seven out of the nine traits showed significant changes under S1 treatments, four out of the nine traits showed significant changes under S2 treatments, and five out of the nine traits showed significant changes under S3 treatment (**Figure 2B**). *O. biennis* had higher TB, LBR, CA, LP, and lower H, LN, LN/LP than *C. album* under S1 treatment (**Figure 2B**). *O. biennis* had higher LBR, CA, and lower H and LN than *C. album* under S2 treatment (**Figure 2B**). Under S3 treatment, *O. biennis* had higher TB, LBR, CA, and lower H, LN/LP than *C. album* (**Figure 2B**).

Eight of the twelve traits (TB, LBR, H, CA, SLA, Fv/Fm, Chl, and LN) had greater effect sizes on *O. biennis* than in *I. japonica* in the S1 treatment; however, RSR followed the opposite direction (**Figure 2C**). Under S2 treatment, eight of the twelve traits (TB, LBR, H, CA, SLA, Fv/Fm, Chl, and LN/LP) had greater effect sizes on *O. biennis* than in *I. japonica*, while RSR and LP followed the opposite direction (**Figure 2C**). Under S3 treatment, four of the twelve traits (TB, CA, SLA, and Fv/Fm) of *O. biennis* were higher than those of *I. japonica*, while RSR was lower than those of *I. japonica* (**Figure 2C**).

### Effect size of mixture plantation on invasive species

**Figure 3:**
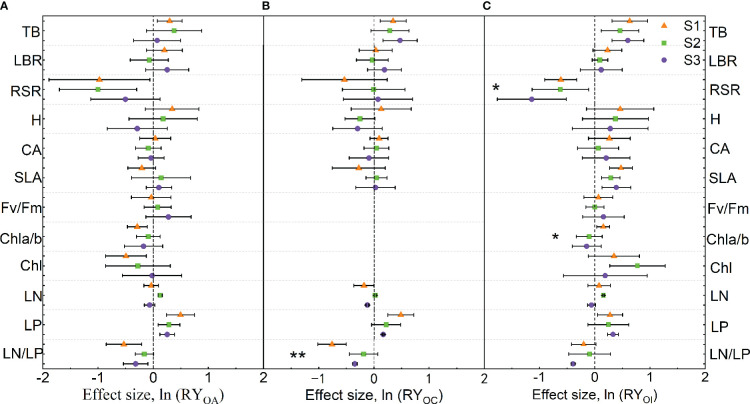
Effect sizes of mixture plantation (biological interaction) with native species (*Artemisia argyi*, *Chenopodium album*, and *Inula japonica*) on invasive species (*Oenothera biennis*) traits in three salinity treatments. OA, *O. biennis* in mixed culture with *A. argyi*; OC, *O. biennis* in mixed culture with *C. album*; OI, *O. biennis* in mixed culture with *I. japonica*. The orange triangle illustrates the S1 treatment (control), the green square illustrates the S2 treatment (low salt treatment), and the purple circular illustrates the S3 treatment (high salt treatment). Error bars are 95% confidence intervals. Significant effect sizes are indicated by confidence intervals that do not overlap zero. The asterisk in the figures indicates significant effects of salt stress on different functional traits of *O*. *biennis* under interspecific interaction. The capital letters represent the traits of *O*. *biennis* when mixed with native species - A. *argyi* **(A)**, *C. album* **(B)**, and *I. japonica* **(C)** and when monoculture. Abbreviations have identical meanings as described in **Table 1**.

When mixed with *A. argyi*, the mixture significantly increased TB and LP of *O. biennis*, while RSR, Chl a/b, Chl, and LN/LP of *O. biennis* were reduced under S1 treatment. The mixture significantly increased LN and LP of *O. biennis*, whereas decreased the RSR of *O. biennis* under S2 treatment. The mixture significantly increased the LP of *O. biennis* and decreased the LN/LP of *O. biennis* under S3 treatment (**Figure 3A**). Salinity had no significant effect on the trait values of *O. biennis* when mixed with *A. argyi* (**Figure 3A**).

When mixed with *C. album*, the mixture significantly increased TB and LP of *O. biennis*, while decreasing LN/LP of *O. biennis* under S1 treatment. There was no significant difference in the traits of *O. biennis* between monoculture and mixture under S2 treatment. The mixture significantly increased TB and LP of *O. biennis* and decreased LN and LN/LP of *O. biennis* under S3 treatment (**Figure 3B**). Salinity significantly increased LN/LP of *O. biennis* when mixed with *C. album* (**Figure 3B**).

When mixed with *I. japonica*, the mixture significantly increased TB, SLA, Chl a/b, and LP of *O. biennis*, while decreasing RSR of *O. biennis* under S1 treatment. The mixture significantly increased TB, SLA, Chl, and LN of *O. biennis*, whereas decreased the RSR of *O. biennis* under S2 treatment. The mixture significantly increased TB, SLA, and LP of *O. biennis* and decreased RSR and LN/LP of *O. biennis* under S3 treatment (**Figure 3C**). Salinity significantly decreased RSR and Chl a/b of *O. biennis* when mixed with *I. japonica* (**Figure 3C**).

### Effect size of mixture plantation on native species

**Figure 4:**
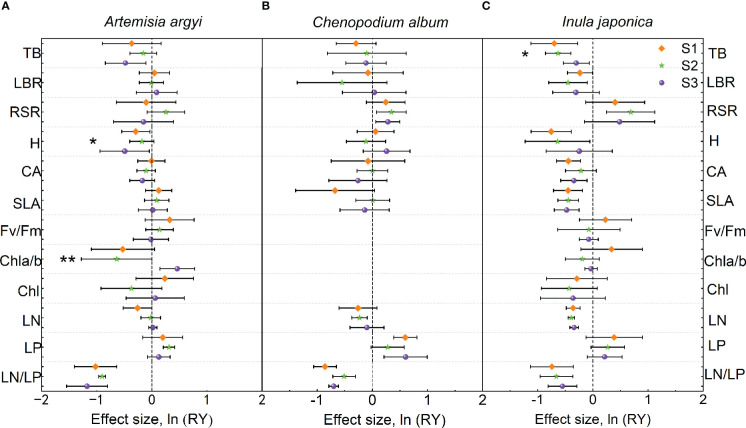
Effect sizes of mixture plantation (biological interaction) with invasive species (*Oenothera biennis*) on three native species (*Artemisia argyi*, *Chenopodium album*, and *Inula japonica*) traits in three salinity treatments. The orange diamond illustrates the S1 treatment (control), the green pentagram illustrates the S2 treatment (low salt treatment), and the purple sphere illustrates the S3 treatment (high salt treatment). Error bars are 95% confidence intervals. Significant effect sizes are indicated by confidence intervals that do not overlap zero. The asterisk in the figures indicates significant effects of salt stress on different functional traits of native species under interspecific interaction. The capital letters represent the traits of three native species - A. *argyi* **(A)**, *C. album* **(B)**, and *I. japonica* **(C)** when mixed with *O*. *biennis* and when monoculture. Abbreviations have identical meanings as described in **Table 1**.

The H and LN/LP of *A. argyi* in the mixture were lower than those in monoculture under S1 treatment; the LP in the mixture was higher than that in monoculture, while LN/LP was lower than that in monoculture under S2 treatment; the Chl a/b in the mixture was higher than that in monoculture, but TB, H, and LN/LP in the mixture were lower than those in monoculture under S3 treatment (**Figure 4A**). Salinity significantly increased Chl a/b but decreased H of *A. argyi* (**Figure 4A**).

The LP of *C. album* in the mixture was higher than that in monoculture, but LN/LP in the mixture was lower than that in monoculture under S1 treatment; the RSR in the mixture was higher than that in monoculture, while LN and LN/LP were lower than those in monoculture under S2 treatment; the RSR and LP in the mixture were higher than those in monoculture, but LN/LP in the mixture was lower than that in monoculture under S3 treatment (**Figure 4B**). Salinity had no significant effect on the trait values of *C. album* (**Figure 4B**).

Numerous traits (TB, LBR, H, CA, SLA, LN, and LN/LP) of *I. japonica* in the mixture were lower than those in monoculture under three salt treatments. However, RSR of *I. japonica* in the mixture was higher than that in monoculture under S2 treatment (**Figure 4C**). Salinity significantly increased the TB of *I. japonica* (**Figure 4C**).

### Effects of plantation and salinity on soil properties

**Figure 5:**
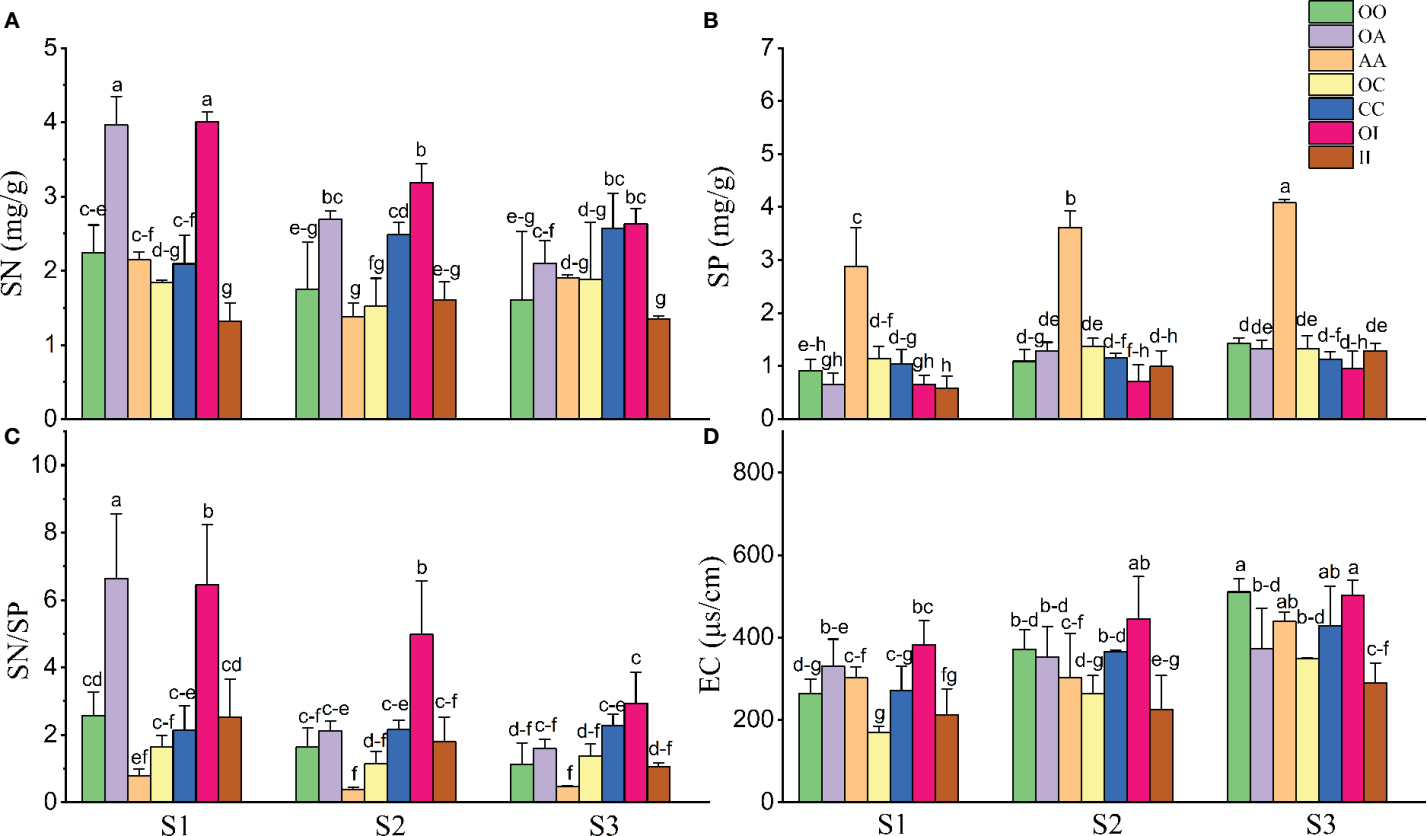
Soil nitrogen concentration (SN) (*n* = 3-5, **A**), soil phosphorus concentration (SP) (*n* = 3-5, **B**), the ratio of soil nitrogen to phosphorus (SN/SP) (*n* = 3-5, **C**), and soil electrical conductivity (EC) (*n* = 3-5, **D**) after the experiment in monocultures and mixtures (OO, *Oenothera biennis* in monoculture; OA, *O. biennis* in mixed culture with *Artemisia argyi*; AA, *A. argyi* in monoculture; OC, *O. biennis* in mixed culture with *Chenopodium album*; CC, *C. album* in monoculture; OI, *O. biennis* in mixed culture with *Inula japonica*; II, *I. japonica* in monoculture) under three salinity treatments. Values are presented as mean ± *SE*. Different lowercase letters denote significant differences at *α* = 0.05 in Duncan’s *post-hoc* test. The lowercase letters on top of the bars are presented as “c-e” and “c-f” means “cde” and “cdef”, respectively.

Overall, salinity increased SP and EC and decreased the SN and SN/SP of both native and invasive plants under both monoculture and mixture (**Table 2**, **Figure 5**). The SN and SN/SP of *O. biennis* in the mixtures of *A. argyi* or *I. japonica* were significantly higher than those in other cultivation among most salt treatments (**Figures 5A, C**). However, the SP in the monoculture of *A. argyi* was higher than other cultivation under all salt treatments (**Figure 5B**).

**Table 2:** Results of two-way ANOVA for the effects of salinity (S), cultivation (C), and their interactions (S × C) on soil properties.

Parameters	Salinity (S)	Cultivation (C)	Salinity × Cultivation (S×C)
SN (mg/g)	11.874^**^	32.864^**^	5.281^ns^
SP (mg/g)	24.413^**^	139.461^**^	2.206^*^
SN/SP	23.798^**^	27.699^**^	5.959^**^
EC (μs/cm)	27.163^**^	11.977^**^	1.123^ns^

SN, soil nitrogen concentration; SP, soil phosphorus concentration; SN/SP the ratio of soil nitrogen to phosphorus, and EC soil electrical conductivity.

Numbers in the table represent F values; asterisks indicate significant effects: ^**^ p ≤ 0.01, ^*^ p ≤ 0.05, and ^ns^ p>0.05.

### Principal component analysis

**Figure 6:**
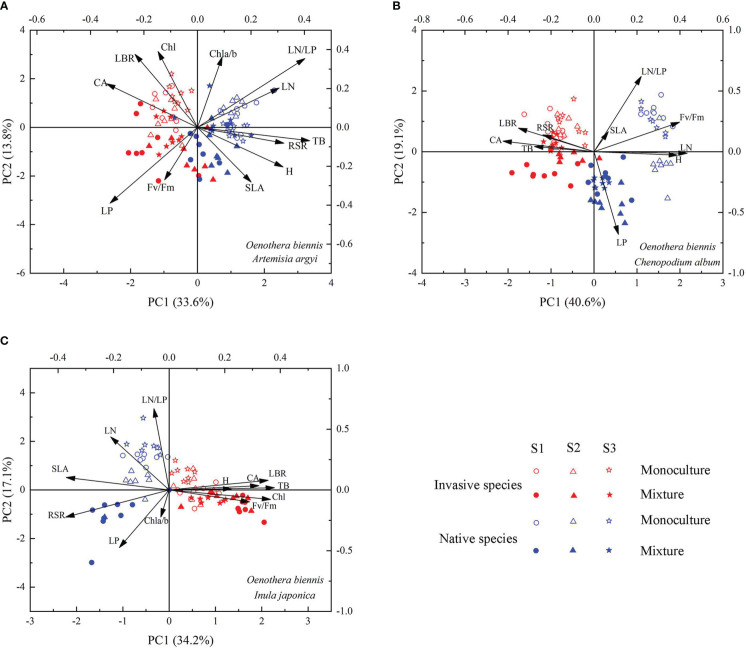
Principal component analysis of *Oenothera biennis* and three native species (*Artemisia argyi*, *Chenopodium album*, and *Inula japonica*) across three levels of soil salinity and two cultivation types based on 12 functional traits. Abbreviations have identical meanings as described in **Table 1**.

The functional traits of invasive species *O*. *biennis* and three native species (*A. argyi*, *C. album*, and *I. japonica*, respectively) were separated along the PC1 axis and the two cultivation types were separated clearly along the PC2 axis (**Figure 6**). When mixed with *A. argyi*, the first axis (with 33.6% of the variation explained) was associated with TB, RSR, H, CA, LN, LP, and LN/LP (**Table S1**; **Figure 6A**). The second axis, accounting for 13.8% of the variation, was mainly affected by LBR, Chl a/b, Chl, and LN/LP (**Table S1**; **Figure 6A**). When mixed with *C. album*, the first axis (with 40.6% of the variation explained) was associated with LBR, H, CA, Fv/Fm, and LN (**Table S1**; **Figure 6B**). The second axis, accounting for 19.1% of the variation, was mainly affected by LP and LN/LP (**Table S1**; **Figure 6B**). When mixed with *I. japonica*, the first axis (with 34.2% of the variation explained) was associated with TB, LBR, RSR, CA, SLA, and Chl (**Table S1**; **Figure 6C**). The second axis, accounting for 17.1% of the variation, was mainly affected by LN, LP, and LN/LP (**Table S1**; **Figure 6C**). The distribution order of invasive species and three native species along the PC2 axis is similar, mainly monoculture and mixture seedlings from positive coordinate to negative coordinate, however, the three salinity treatments were not separated clearly (**Figure 6**).

## Discussion

In this study, we examined the performances and competition interactions between the invasive species (*O. biennis*) and three native species (*A. argyi*, *C. album*, and *I. japonica*) at three salinity levels. The result of our study showed that the total biomass and biomass-based RDI of *O. biennis* were significantly higher than that of *C. album* and *I. japonica*, but lower than *A. argyi*, regardless of the soil salinity. The results answered our first question and concluded that *O. biennis* is not generally a superior competitor to the three native species. The competitive dominance of *O. biennis* is largely associated with the species of native plants, rather than soil salinity. However, salinity influenced the intensity of competition between *O. biennis* and three native species. Salinity alleviated the competitive effect of *O. biennis* on *I. japonica*, but not on the other two native species. Therefore, the invasive species is not always superior competitors in competition with different native species, and the intensity of competition rather than competition outcome between *O. biennis* and three native species varied with the salinity.

### Competition outcomes between *O. biennis* and three native species

At all salinity levels, the total biomass and biomass-based RDI of *O. biennis* were significantly higher than that of *C. album* and *I. japonica*, but lower than *A. argyi*. The results suggested that *O. biennis* is a superior competitor when mixed with *C. album* and *I. japonica*, but not when mixed with *A. argyi*, at least for the population used under the particular conditions of this study for the traits measured. Therefore, we concluded that *O. biennis* could outcompete some, but not all, native species.

Plant functional traits play a key role in the competition between invasive and native species, including growth rate, biomass allocation, SLA, and photosynthetic traits (Wang et al., 2018; Rathee et al., 2021). In our study, most functional traits of *O. biennis* were significantly different from *C. album* and *I. japonica* suggesting that our results support the phenotypic divergence hypothesis rather than the phenotypic convergence hypothesis (Ordonez et al., 2010; Guo et al., 2020), meaning that *O. biennis* has significantly advantageous traits compared to the native *C. album* and *I. japonica*. According to the results of PCA, invasive species *O. biennis* and two native species *C. album* and *I. japonica* were clearly separated, and besides, the differences in most trait values were different, which also supported the phenotypic divergence hypothesis (Ordonez et al., 2010).

When mixed with *C. album*, the competitive advantage of *O. biennis* was mainly attributed to high LBR and CA. Allocation of more biomass to aboveground parts allows invasive *O. biennis* to compete more effectively for light, which helps *O. biennis* to compete successfully with *C. album*. This was consistent with the balanced growth hypothesis, which suggests that plants may increase the biomass allocated to organs involved in resource acquisition when faced with limited resources (Luo et al., 2014). The larger canopy of *O. biennis* may also contribute to superior competitiveness for light. Although *C. album* is an annual species that has a fast life history and is taller than *O. biennis*, it still cannot out-compete *O. biennis*.

When mixed with *I. japonica*, *O. biennis* had higher LBR, H, CA, SLA, Fv/Fm, and Chl and lower RSR, indicating that *O. biennis* obtained more light energy and accumulated more biomass through aboveground traits, and outcompeted *I. japonica*. Invasive plants often adopt different biomass allocation strategies in response to environmental changes to create a competitive advantage for themselves (Guo et al., 2020). Allocating more biomass from roots to aboveground parts, *O. biennis* obtained a higher height and a large canopy than *I. japonica*, which contributed to capturing light effectively and inhibited the growth of *I. japonica* through shading (Luo et al., 2014). Additionally, photosynthetic capacity is closely related to chlorophyll content and chlorophyll fluorescence parameters. *O. biennis* had higher Fv/Fm and Chl than *I. japonica*, meaning that it tends to synthesize a greater quantity of chlorophyll to improve photosynthetic capacity, thereby enhancing its performance (Guo et al., 2019).

Specific leaf area is a key leaf trait closely related to plant growth and survival strategies (Legault et al., 2018). Our results showed the SLA of *O. biennis* was higher than that of *I. japonica*, which is consistent with the results of previous studies that high SLA promotes invasiveness (Dawson et al., 2012; Hajiboland et al., 2020). SLA is a basic defensive trait associated with leaf toughness and is negatively correlated with cell wall quality (Feng et al., 2008). A higher SLA of *O. biennis* than *I. japonica* may help *O*. *biennis* allocate more resources to photosynthesis and increase light capture, thereby enhancing plant performance in fertile environments.

Except for competitive exclusion, some native species can coexist with invasive species and even dominate the plant community (MacDougall et al., 2009). In this study, most functional traits of *O. biennis* were not significantly different from *A. argyi* and the total biomass of *O. biennis* was lower than that of *A. argyi* under S2 and S3 treatments, suggesting that more similar traits were observed between *A. argyi* and *O. biennis*, and *A. argyi* is more competitive than *O. biennis*. Our results support the limiting similarity hypothesis that native species in the community should inhibit the invasion of functionally similar species (Emery, 2007).

### The intensity of competition between *O. biennis* and three native species

The strength of interspecific competition refers to the ability of a species to tolerate and inhibit other species (Zhang and van Kleunen, 2019). The strength of interspecific competition is often used as a substitute for competitive outcomes, in which species will exclude or dominate over other species in the same community (Gibson et al., 1999). In our study, the mixture significantly increased TB of *O. biennis* under S1 and S3 treatments when mixed with *C. album*, while TB of *C. album* grown in the mixture with *O. biennis* was not different from that of *C. album* grown in the monoculture in three salt levels. This indicates the interspecific competition promoted the growth of *O. biennis*, however, *C. album* was not affected by *O. biennis* during the experiment. Mixture significantly increased TB of *O. biennis* and decreased TB of *I. japonica* in all salt treatments, suggesting that interspecific competition promoted the growth of invasive *O. biennis* and suppressed the growth of native *I. japonica*. However, when mixed with *A. argyi*, the mixture only significantly increased TB of *O. biennis* under the S1 treatment but did not change it under the S2 and S3 treatments. The TB of *A. argyi* in the mixture was not different from that in monoculture under the S1 and S2 treatments, while was lower than that in the monoculture under the S3 treatment. In the face of interspecific competition, *O. biennis* adopted a positive or even maintenance strategy, while *A. argyi* adopted maintenance or a negative strategy. Thus, although the intensity of interspecific competition may be a critical determinant of competitive outcomes, the two measures are not equivalent (Weigelt and Jolliffe, 2003; Zhang and van Kleunen, 2019).

Under competitive pressure from *O. biennis*, *C. album* and *I. japonica* partitioned more biomass to roots than those in monocultures, while the RSR of *A. argyi* did not change significantly. This may be because that *C. album* and *I. japonica* have rhizomes, which can absorb more nutrients and water. Our results supported the root aggregation theory that plants may gain or maintain exclusivity to limited resources through root proliferation and root aggregation as a defensive response to resist invasion (Yuan et al., 2013). However, the RSR of *O. biennis* followed the opposite direction when mixed with *A. argyi* and *I. japonica*, indicating that *O. biennis* was able to capture light effectively by allocating more biomass to aboveground parts. Moreover, the mixture significantly increased the SLA of *O. biennis* when mixed with *I. japonica*, indicating that *O. biennis* reduced the allocation of defense structures and increased the allocation of photosynthetic tissues, helping it increase light capture and thus maintaining a high biomass production (Luo et al., 2014). Compared with monoculture, the resource acquisition traits (LBR, H, CA, and SLA) of *I. japonica* were significantly reduced in the mixture, which may be one of the reasons why *I. japonica* was inhibited by interspecific competition.

Many studies found that leaf nitrogen concentration is positively correlated with plant photosynthesis capacity (Luo et al., 2014; Wang et al., 2022). In our study, the higher leaf nitrogen concentration of *O. biennis* mixed with *A. argyi* and *I. japonica* may be interpreted as a matter of greater photosynthetic rate in the mixture than that in the monoculture at low salt addition levels. This result was also related to the increase of soil nitrogen concentrations when *O. biennis* was mixed with *A. argyi* and *I. japonica*, and more nitrogen could be allocated to photosynthetic capacity under an abundant soil nitrogen supply. The afore-stated result demonstrated that higher leaf nitrogen concentration is favorable for the invasion of alien species, which was consistent with previous research results (Yuan et al., 2013; Wang et al., 2022). Regardless of mixing with native species, *O. biennis* had higher LP and lower LN/LP than those of monoculture, suggesting that *O. biennis* could invest more P in leaf photosynthetic apparatus, thus contributing to the capture and absorption of light resources. Therefore, this strong ability to absorb the resources of invasive species may provide an advantage for their successful invasion.

### Effects of salt stress on the biological interactions between *O. biennis* and three native species

In the present study, the EC of the soil changed greatly due to the addition of salt, suggesting that the salt treatment had successfully created three salinity levels. However, the RDI of *O. biennis* did not change significantly among various salt levels, indicating that increased salinity did not significantly alter the competitive dominance of *A. argyi* over *O. biennis* and *O. biennis* over *C. album* and *I. japonica*. Furthermore, based on the results of PCA, the three salinity treatments were not separated clearly. Therefore, our results demonstrated that increased salt stress did not change the competitive outcome between invasive and native species.

Numerous studies have also confirmed that salinity has detrimental effects on plant growth and development, leading to a reduction of biomass (Guo et al., 2018; Meyerson et al., 2020; van Zelm et al., 2020; Sogoni et al., 2021). However, in this study, salinity had no significant effect on the total biomass of *O. biennis*, regardless of which native species was mixed with it. Our results did not support the stress gradient hypothesis, that is, interspecific interactions tended to facilitate as salt stress increased (Bertness and Callaway, 1994). One possible explanation is that the amount of salt added in different salt treatments was not sufficient to cause shifts in interspecific competition, and/or the short duration of the experiment. *Oenothera biennis* usually occurs in open and disturbed sites, such as river banks and coastal areas, thus having certain salt tolerance. However, the salt levels we set may be too low to cause shifts in the biomass of *O*. *biennis*. Alternatively, short-term addition of salt may not expose *O*. *biennis* to osmotic stress. In terms of native species, the total biomasses of *A. argyi* and *C. album* were also not affected by salinity when mixed with *O. biennis*, because that *A*. *argyi* and *C*. *album* have certain salt tolerance. *Chenopodium album* is a halophytic plant species that tolerates salt stress by increasing redox potential associated with a large number of osmolytes and antioxidants (Tanveer and Shah, 2017). The negative impacts of *O. biennis* on *I. japonica* were alleviated because in the mixture the total biomass of *I. japonica* significantly increased while that of *O. biennis* remained unchanged with increasing salinity. This result suggested that low resource availabilities would increase the relative competitive ability of the native due to a negative relationship between salinity and the availability of water or nutrients (Kolb and Alpert, 2003).

Salt stress generally reduces root allocation and modifies the distribution of different root systems (Mao et al., 2016). The RSR of *O. biennis* decreased with increasing salinity when mixed with *I. japonica*, which was associated with the severe reduction of root biomass. This may be caused by the accumulation of soluble salts in the root zone, which limits water uptake by plant roots and results in osmotic stress (Sogoni et al., 2021). Previous studies have shown that salt stress reduced chlorophyll content in leaves through the degrading of chlorophyll structure (Baniasadi et al., 2018). Chlorophyll a is associated with the photosynthetic reaction centers and chlorophyll b with the light-harvesting complex (Guo et al., 2018). The Chl a/b of *O. biennis* decreased with increasing salinity when mixed with *I. japonica*, which could be interpreted as a higher investment in the light-harvesting complex and required to cope with salt stress by capturing more light.

Plants obtain mineral nutrients, including nitrogen (N) and phosphorus (P) from the soil, and nutrient cycling is affected by the accumulation of salts in the soil (Miura, 2013). Our results showed that salinity significantly reduced the SN and SN/SP of *O. biennis* when mixed with *A. argyi* and *I. japonica*. Salt stress interfered with biological fixation and uptake of nitrogen, which may reduce soil N sources (Farooq et al., 2017; Sogoni et al., 2021). In addition, soil salinity can also alter nutrient recycling by influencing soil microorganisms (Rath and Rousk, 2015). High salinity may cause hypoxia in plant roots and increase the activity of denitrifying bacteria, which fixed N reenters the atmosphere as N_2_, leading to the reduction of soil nitrogen (van Zelm et al., 2020; Ding et al., 2021). Phosphorus, another important indicator of soil nutrient status, can be used to regulate some enzymes (phosphorylation), energy transfer, and carbohydrate transport (Miura, 2013; Shi et al., 2021). Soil phosphorus content of *O. biennis* increased with increasing salinity in monoculture and mixed, which may be because salinity improves the activity of phosphatase on plant roots, thus increasing phosphorus content in the soil (Miura, 2013).

## Conclusions

Generally, the success of invasive species is mainly attributed to their high competitive dominance. However, our study has demonstrated that invasive species are not always superior competitors in competition with different native species. In competition with *I. japonica*, *O. biennis* obtained a greater competitive advantage by increasing LBR, H, CA, SLA, Fv/Fm, and Chl and decreasing RSR to capture and absorb more resources. When mixed with *C. album*, native species *C. album* was not threatened by the invasive species *O. biennis*, although *O. biennis* was a superior competitor and grew well in the mixture. In contrast, when competing with *A. argyi*, *O. biennis* was not a superior competitor because *A. argyi* has higher biomass than *O. biennis*, resulting in that *O. biennis* being less competitive than *A. argyi*. With the increase in salinity, the competitive effect of *O. biennis* on *I. japonica* was alleviated, but the competitive effects between *O. biennis* and the other two native species were not affected by salinity. Salinity did not alter the competitive outcome between invasive and native species. However, our experiment was conducted in the greenhouse, which cannot simulate complex natural environments, and the seedlings were treated with salt stress for 2 months, so our results should not be extrapolated arbitrarily to field conditions. Therefore, further studies with field experiments or more long-term experiments are necessary to better understand the invasion mechanisms of *O. biennis* under global change scenarios.

## Data availability statement

The original contributions presented in the study are included in the article/**Supplementary Material**. Further inquiries can be directed to the corresponding authors.

## Author contributions

XG and K-LW conceived, designed, and supervised this research. HW did the experiments and collected data. J-YM analyzed the data. J-YM and XG wrote the paper. L-LL, M-YL, Y-KS, TW, and LM commented on previous versions of the manuscript. All authors contributed to the article and approved the submitted version.
