# Supplementary material for: Effects of salt stress on interspecific competition between an invasive alien plant Oenothera biennis and three native species
Source: Front Plant Sci. 2023 Mar 21;14:1144511. doi: 10.3389/fpls.2023.1144511 (PMC10070839; doi:10.3389/fpls.2023.1144511)
Supplement: Supplementary file 1 [file DataSheet_1.pdf]

Table S1. Loadings in the principal components analysis (PCA) of plant functional traits

Species	*Oenothera biennis* vs. *Artemisia argyi*		*Oenothera biennis* vs. *Chenopodium album*		*Oenothera biennis* vs. *Inula japonica*	
Abbreviation	PC1	PC2	PC1	PC2	PC1	PC2
TB	**0.420**	-0.069	-0.277	0.043	**0.379**	0.018
LBR	-0.234	**0.377**	**-0.352**	0.194	**0.357**	0.079
RSR	**0.323**	-0.081	-0.238	0.138	**-0.373**	-0.225
H	**0.320**	-0.204	**0.380**	-0.029	0.226	0.009
CA	**-0.340**	0.225	**-0.423**	0.087	**0.323**	0.035
SLA	0.201	-0.281	0.067	0.165	**-0.371**	0.102
Fv/Fm	-0.123	-0.270	**0.398**	0.243	0.290	-0.099
Chl a/b	0.092	**0.362**			-0.030	-0.225
Chl	-0.147	**0.390**			**0.365**	-0.079
LN	**0.305**	0.203	**0.434**	-0.013	-0.210	**0.437**
LP	**-0.327**	**-0.390**	0.114	**-0.686**	-0.179	**-0.474**
LN/LP	**0.402**	**0.354**	0.220	**0.619**	-0.055	**0.670**

Bold indicates absolute values > 0.30, which was regarded as key factor. Abbreviations have identical meanings as described in Table 1.
